# Institutional Questions

**Published:** 1899-11-11

**Authors:** 


### INSTITUTIONAL QUESTIONS.

### The Cost of a Hospital.

"M. O." writes: I shall be much obliged if you will give me some information *re* the cost per bed of building a hospital. It is proposed to erect one for seamen, having accommodation for sixty patients. Provision must be made for quarters for the resident medical officer and his family. It is probable that no women nurses will be employed. An out-patient department must be provided.

*** The cost of building a hospital depends naturally very much upon whether it is to be in London or the country. If in London, such a hospital as you describe could be erected for about £300 per bed. If in the country the cost would be certainly 25 per cent. less. The elimination of one sex would, of course, make the planning less expensive and elaborate.

### Portable Hospitals.

M. O. H. writes: I shall be glad if you can advise me on the following point. At times our accommodation for infectious cases proves hardly equal to the demand upon it, but, as this is only an occasional difficulty, it is not thought necessary to add for the present to our permanent buildings. Can you recommend me some form of temporary building which might be used in emergencies and lend itself without difficulty to the purposes of a sick ward?

*** To supply extra accommodation in times of great pressure nothing would answer your requirements more efficiently than the excellent little portable "Berthon" hospital huts. At the Bournemouth Sanitary Hospital they are found to work well for a similar purpose, and two or three have been in use there for several years with great success. Dr. Nunn, the medical officer of health for Bournemouth, has given them his unqualified approval, finding them to be weather-tight, easily regulated in the matter of temperature, and with a system of ventilation "not to be improved on, and most efficient." Very welcome assistance was afforded by Bournemouth at the time of the Maidstone epidemic of typhoid, when three of these huts were sent from the Corporation, and put up in the grounds of the isolation hospital. Cases nursed in them did well, and much satisfaction was expressed at their efficiency. You can get full particulars by writing to the Berthon Boat Company, Limited, Romsey, Hampshire.

